# Alveolar Abscess

**Published:** 1877-09

**Authors:** J. A. Turner


### ARTICLE V.

### *Alveolar Abscess.*

BY J. A. TURNER.

Read before the Indiana State Dental Society.

The frequency of this affection renders it one of great importance to the dentist. It has received the earnest consideration of the dental profession for many years.

I do not now expect nor aim to present anything new upon the subject, but rather to direct the attention to the old and reliable methods that have been proved.

That an alveolar abscess can form and continue in a state of considerable activity, and that without causing pain or discomfort, especially after its formation, sometimes for years, is a very interesting fact, one which, if well studied, will teach an important lesson.

First, then, we shall take the ground that there can be no permanent results attained in the cure of alveolar abscess until we can control the patient. If the only questions were, "can I treat locally an alveolar abscess, and save the tooth to years of usefulness?" it were a simple question and easily answered. But this would be stopping short of our goal, and would degrade dentistry to a level with the tinner and gunsmith, with nothing to do but to mend and patch, and patch and mend.

In order to control patients, we should have the broadest knowledge of our profession possible, taking in all that the general practitioner of medicine is supposed to know, and also possess a liberal knowledge of our specialty. If we wanted to set bounds to this condition, (disease) we should say to the scrofulous parent: "Stop propagating your kind in the world." To the syphilitic parents, that you stop bringing into this bright, beautiful world, offspring that will be very susceptible to many of the worst ills that flesh is heir to, and so on through the catalogue. It would be an exception, I was going to say an anomoly to see parents with some such diatheses, bring forth children whose teeth would be perfect and able to resist the encroachments of decay, and so the occurrence of abscess.

We talk sometimes loudly about graham flour, and beans and potatoes, and go through a long list of dietary articles, which our patients should use, and we do well, for these are quite as necessary as that, after the boiler is built there should be the application of water and fuel, that it may be made to drive the engine and so fill its mission. But you see how trifling it would be to pay little or no attention to the building of the boiler, and merely lay in a good supply of first-class fuel and plenty of water, when perhaps the first time any great strain were made upon it, it would give away and utterly fail. We can no more expect to cure this condition by dietary and habits of life, where the seeds of disease are shown by parents, than you could to make the boiler fill its mission by the best of bituminuous coal, and the purest of rain water. I am not claiming now that individual cases cannot be cured. One swallow does not make a summer, and the cure of an individual case does not prevent the next one. This is not getting down to a hard pan. This would not be digging to a solid foundation and raising a substantial structure. Like father like child. Like begets like. A law that approaches the Medes and Persians in its unbending aud unyielding nature. If these premises then be correct, we cannot long remain in doubt as to the cure. It may be true that if a sound and healthy person were compelled to reside in a malarious country upon unwholesome food, scantily doled out, he would soon deteriorate. But these are extraneous causes and not the causes *per se.* Two hundred years ago, when French missionaries were trying to win the nations of our soil to become French, they were struck by the muscular developments and robust constitutions of both male and female. Indeed there were no cripples, no sick, no diseased. Why? Because the savage life required warriors and men and women who could endure fatigue and hardships, and when one became crippled or deformed or sick, they were left in the wilderness to starve and die, so the timber of which they were built stopped with them.

In the study of disease it is an argument against civilization that man is allowed to propagate his species, knowing that he will entail misery and bring ruin to his race by imparting the seeds of weakness and disease. He fills hospitals and poor houses and grave yards, and brings to an untimely end his own creation. There is but one compensation for all this, that it brings out the god like in our characters, and eleemosynary establishments and homes for the helpless are built and endowed by man.

As to the causes and cures of individual cases, we have enough food for careful reflection. Among the causes, we may maintain periostitis, or exposure of the pulp, forcing of cotton, gold, or the broken point of an instrument through the apex of the root, sudden stroke or blow to the tooth, thermal changes in cases of abrasion, and many others. Where the pus has not yet formed vents, it were best to open through root if possible, otherwise through gum. Too much care cannot be given to breaking up the sac and bringing about a healthy condition. Where apex of root is open sufficiently large, pumping is best.

It may be asked when the condition justifies filling? Most of our authors, I believe, say whenever the part has healed up. I think this unnecessarily prolongs the case without adding anything to the certainty of the result. I should say from experience, that whenever you can get a secretion from the part that resembles serum, or is even mixed with blood, finish your work. I have tried the various materials for filling the roots and find that none works so well as gold. In some cases the walls of the canals seem to be left in a hyper-sensitive condition and Os Artificial produces too much pain to be endured.

Hill's stopping I have never satisfied myself would not become softened, and so carried out in cases of fistulous openings through gums. Pack at apex, a pleget of cotton with carbolic acid or creosote and fill with gold, and you have the best filling possible known to us now.—*Dental Register.*

